# Efficacy and Safety of Sofosbuvir and Ribavirin in an Italian Cohort of HCV Genotype 2 Elderly Cirrhotic Patients

**DOI:** 10.5152/eurasianjmed.2022.20421

**Published:** 2022-06-01

**Authors:** Carlo Smirne, Roberto Carbone, Cosimo Colletta, Paolo Scivetti, Pier Paolo Sainaghi, Grossini Elena, Mario Pirisi

**Affiliations:** 1 Department of Translational Medicine, Università del Piemonte Orientale UPO, Novara, Italy; 2 Internal Medicine Unit, AOU Maggiore della Carità Hospital, Novara, Italy; 3 Gastroenterology and Infectious Diseases Units, SS Antonio e Biagio e Cesare Arrigo Hospital, Alessandria, Italy; 4 Internal Medicine Unit, Madonna del Popolo Hospital, Omegna, Italy; 5 Internal Medicine Unit, Degli Infermi Hospital, Ponderano, Italy

**Keywords:** Hepatitis C, cirrhosis, sofosbuvir, ribavirin, duration of therapy, sustained virologic response

## Abstract

**Objective:** Sofosbuvir and ribavirin represented until recently the standard of care in hepatitis C virus genotype 2 cirrhotic patients. In registration trials, 12-16 week durations were associated with 90% sustained virological responses, although not confirmed by real-life studies. In Italy, various durations (12,16, 20, and 24 weeks) represent lawfully reimbursable healthcare practice. The aim is, therefore, to study the behavior of Italian clinicians and the possible impact of therapy durations on sustained virological responses and patient safety.

**Materials and Methods:** Data of all consecutive genotype 2 cirrhotic patients who started sofosbuvir plus ribavirin therapy between January 2015 and March 2017 in 7 Italian liver clinics were collected retrospectively.

**Results:** Overall, 147 patients (138 Child–Pugh A, mean age: 71 years) were treated. The median treatment duration was 16 weeks, but marked differences were found among the clinicians; however, the 12-week duration was not considered by the vast majority of them. Rates of intention-to-treat and per-protocol sustained virological responses were 95.9% and 97.1%, respectively, and neither showed differences between the various durations. No independent, sustained virological response predictors could be found, but the median baselines for Child–Pugh and Model For End-Stage Liver Disease scores were higher in non-responders. Anemia was not associated with treatment duration. One case of acute kidney injury attributed to the possible sofosbuvir effect was reported.

**Conclusion:** In genotype 2 cirrhotic patients, sofosbuvir plus ribavirin was associated with real-life-sustained virological response rates of almost 96%, without a significant impact on treatment duration provided it was longer than 12 weeks.

**Main points**

- Sofosbuvir in combination with ribavirin has been the first all-oral direct-acting antiviral (DAA) to be placed on the market to treat hepatitis C virus (HCV) and opened a new era in the treatment of this global infection.
- The treatment duration of 16-20 weeks was recommended by most guidelines for HCV genotype 2 patients, however, without exact indications on what was the best treatment schedule for subjects with advanced fibrosis/cirrhosis.
- So, nowadays, the exact duration of therapy in this specific subpopulation remains undefined, and also real-life experiences could not provide definitive results.
- We demonstrated that using any recommended extended duration (i.e. >12 weeks) of sofosbuvir/ribavirin in HCV genotype 2 patients with well-documented cirrhosis, 96% of these subjects can be cured with a good safety profile.
- Although this regimen has recently been replaced by more potent DAA combinations as the standard of care for treating HCV genotype 2 patients, our findings may be useful for clinicians from countries where these new regimens are not yet fully available.

## Introduction

All-oral, direct-acting antiviral (DAA) drug combinations have shown high efficacy rates, favorable side-effect profiles, and easy applicability in the treatment of chronic hepatitis C virus (HCV) infection. The combination of sofosbuvir (SOF) and ribavirin (RBV) was historically the first all-oral regimen recommended by the American Association for the Study of Liver Diseases,^1^ and thereby approved by many national health care services.

For what concerns the Italian National Health System, DAA treatments were initially granted only to patients with more advanced liver disease. Things changed when treatments become unrestricted to all HCV-infected patients and when SOF plus RBV scheme began to be replaced by more powerful combinations, such as the fixed combinations of SOF/velpatasvir or glecaprevir/pibrentasvir. However, the latter considerations currently apply only to countries with relatively high gross domestic products, while it is noteworthy that restricted access to newer therapies persists in many other nations, due to their high costs. Therefore, interest remains in these treatments, which are not only historical.

Moreover, for issues related to genotype (GT) 2 infection specifically, we are particularly intrigued because, in our opinion, the current recommendations may still leave room for some clinician discretion, probably resulting from a lack of unanimous consensus in the available medical literature. This area of residual uncertainty applies, in particular, to patients with cirrhosis. The problem is that, although everyone agreed that SOF plus RBV treatment was the best first-line treatment option for these subjects, the main guidelines differ on the ideal duration of this therapy and do not provide univocal indications to the clinicians.

American and Italian guidelines have recommended a 16-week treatment for all cirrhotic patients, while the European and Asian-Pacific guidelines have allowed for treatment extensions out to 20 and 24 weeks, respectively, especially when the subjects were pegylated-interferon (PEG-IFN) experienced. This was in accordance with the SOF data sheet, which recommends a treatment duration of 12 weeks for all HCV GT2-infected patients but points out that consideration should be given to potentially extending the duration of therapy up to 24 weeks for those subgroups of patients who have one or more factors historically associated with lower response rates to IFN-based therapies.^1-3^

In essence, many clinicians could (and can) lawfully administer 4 different treatment schedules to the same patient, namely 12, 16, 20, or 24 weeks. The rationale for these recommendations derived mainly from 5 main phase III clinical trials, none of which, however, was focused specifically on the subpopulation of HCV GT2-infected cirrhotic patients.^4-7^ Globally, these studies showed sustained virological response (SVR) rates around 83% for the 12-week and 16-week durations (higher in naïve subjects) and 100% for the 24-week duration; the 20-week duration was not tested. Altogether, these studies demonstrated that the SOF plus RBV combination—despite having many advantages—was suboptimal in this patient setting, at least for the 12-week duration, probably because it was not adequately studied (collectively, only 49 HCV mono-infected subjects).

There are many other smaller phase III or real-life studies available now. However, most of them are continuing to test with a therapy duration of only 12 weeks, and they have not confirmed the data of the first trials substantially.^8-16^ To the best of our knowledge, only a multi-center real-life experience has been published so far that also tested the 20-week duration and reported an SVR rate of 91%.^17^ According to all available data, no significant efficacy differences have been found between the 12-week and 16-week durations, while prolonging therapy to 20 or 24 weeks was associated with an effectiveness gain of roughly 10%.

Taking into account the aforementioned possible lack of validation on large numbers of SOF plus RBV treatment with regard to the subset of GT2 cirrhotic patients, our primary aim was to characterize the behavior of prescribing physicians in a significant Italian macro-area, with particular focus on treatment durations and RBV management. Secondary endpoints included SVR rates 12 weeks after the end of treatment (SVR12) for each treatment schedule—evaluating whether certain clinical or demographic characteristics of patients may affect the SVR rate—and analysis of the safety profile of the different treatment durations.

## Materials and Methods

Data of all consecutive GT2 cirrhotic HCV-infected patients who started therapy with SOF (Sovaldi®, Gilead Sciences, Carrigtohill, Ireland) plus RBV (Teva B.V., Haarlem, Netherlands) between January 1, 2015, and March 31, 2017, were retrospectively and anonymously collected. When starting treatment, all subjects did not have hepatocellular carcinoma or had a complete response to surgery or locoregional therapies, in accordance with National Health Authorities. The database was closed on January 30, 2018, when the last patient ended 24 weeks of follow-up (FUP) after the end of treatment. Seven North Italian liver disease outpatient clinics participated in this study, representing a minor but significant proportion of the total number of patients treated in the whole region (Piedmont): 2134/4435, 48.1%, in line with the estimated served total population: 2 069 302/4 363 916, 47.4% (source: Italian Ministry of Health). All patients received a body weight-adapted dose of RBV and SOF 400 mg once daily.

All the procedures followed were in accordance with the ethical standards of the Helsinki Declaration of 1975, as revised in 2000. The study protocol was approved by the institutional ethical committee (Comitato Etico Interaziendale Novara, IRB code CE 34/17).Written informed consent was obtained from all individual participants included in the study.

### Statistical Analysis

Continuous variables were expressed as means, medians and ranges, and categorical variables as percentages. The Mann–Whitney, Wilcoxon, and Kruskal–Wallis tests were used to compare continuous non-parametric variables, as appropriate. The Student’s *t*-test was used to compare means with a normal distribution. Pearson’s chi-squared test was used to determine whether there was a significant difference between the expected and the observed frequencies in one or more categories. If sample sizes were small, Fisher’s exact test was preferred in the analysis of contingency tables. The Freeman–Halton extension of the Fisher exact probability test was used for 2 rows by 3 columns contingency tables. Multivariable analyses of SVR were performed with logistic regression using Firth penalized maximum likelihood estimation of the effect of a covariate of interest with adjustment for age and sex. A *P* value of <.05 was considered to be significant. All analyses were performed using Statistica 10.0 statistical software (Statsoft, Palo Alto, Calif, USA).

## Results

### Patients

Subject distribution among GTs was as follows: 1241 (GT1, 58.2%), 343 (GT2, 16%), 378 (GT3, 17.7%), and 172 (GT4, 8.1%). Among GT2, 132 patients were METAVIR F3; of the remaining 211 cirrhotic subjects, 147 were treated with SOF plus RBV and 64 with SOF plus daclatasvir (the latter regimen, as an alternative available in Italy at that time in selected patients).

All of the subjects considered in this study were caucasian HCV mono-infected with native livers. Cirrhosis was defined by histology and/or transient elastography. Hepatitis C virus-RNA levels were measured using ABBOTT RealTime assay with a lower limit of quantitation (LLQ) of 12 IU/mL and a lower limit of detection of 10 IU/mL (Abbott Laboratories, Abbott Park, Ill, USA). Hepatitis C virus genotype was determined by using Innolipa 2.0 assay VERSANT HCV Genotype 2.0 Assay (LiPA) (Siemens Healthcare, Erlangen, Germany). The main characteristics of these 147 patients when starting treatment are presented in Table 1. Anemia was defined according to World Health Organization criteria^18^ and renal function according to National Kidney Foundation guidelines.^19^ Eight subjects (3 males and 5 females) were both anemic and with an estimated glomerular filtration rate of (eGFR) <60 mL/min. Thirty-nine patients had platelets count < 100 ×10^9^/L and 25 albumin < 3.5 g/dL.

### Antiviral Therapy

Tables 2 and 3 show the most salient data about the clinical course of patients during and after SOF plus RBV treatment.

The most common mild adverse events (AE), occurring around 20%, were—as expected—fatigue, nausea, insomnia, and headache. One patient died during antiviral therapy at therapy week (TW) 14 due to rapidly progressive liver failure. He had advanced disease at the beginning of antiviral therapy, with Child–Pugh and Model For End-Stage Liver Disease (MELD) scores of 8 and 11, respectively; HCV-RNA became undetectable from TW4. One other patient discontinued antiviral therapy at TW5 due to rapidly progressive renal failure. He was cryoglobulin-negative and with baseline normal renal function (creatinine: 0.95 mg/dL and eGFR: 78 mL/min); hemoglobin was 12.1 g/dL. Initial RBV dosage was 1200 mg/die (10 mg/kg/die). In the next weeks, creatinine gradually deteriorated (1.49, 2.19, and 2.46 mg/dL at TW2, TW4, and TW5 respectively) and anemia worsened (nadir hemoglobin at TW5: 8.6 g/dL): RBV was reduced to 1000 (TW2) and 600 mg/die (TW4). Both renal ultrasound and urinary sediment were normal, and HCV-RNA became detectable below the LLQ at TW4. At TW5, it was decided to definitively suspend both RBV and SOF. Within 4 weeks, renal function returned to pre-therapy values, but HCV-RNA relapsed. This was the only reported serious AE possibly attributable to SOF.

The most common observed AE was—by far—anemia. Five other subjects managed to complete the planned treatment with SOF but discontinued all RBV within TW5 due to clinically significant anemia (3 grade 2 and 2 grade 3). None of them had baseline hemoglobin < 12 g/dL or eGFR < 60 mL/min, but 3 were women and all were over 75 years of age. Two patients could restore reduced RBV dosage; the other ones continued with SOF monotherapy. All other 140 patients managed to complete the entire cycle of SOF and RBV therapy, although 60 of them had to at least temporarily reduce RBV (minimum reported dosage achieved: 200 mg/die). In our casuistry, the onset of anemia was not associated with the duration of treatment.

According to patient grouping by weight, the median dosages of RBV used were 1000 mg (<75 kg, n = 91) and 1200 mg (≥75 kg, n = 56), with respect to the SOF data sheet. However, only 84 (57%) patients received the exact RBV daily dosage as recommended. More in detail, RBV initial doses were respectively in patients <75 kg or ≥75 kg: 600 mg (n = 4), 800 mg (n = 31), 1000 mg (n = 56); 1000 mg (n = 27), 1200 mg (n = 27), and 1400 mg (n = 2). When normalizing RBV to patient weight, median RBV dosage was higher in patients <75 kg (*P* < .001).

In our study population, mean Child–Pugh and MELD scores did not show a significant improvement at the end of treatment (ET) and at FUP at 12 and 24 weeks. No statistical significance was reached also in a per-protocol (PP) analysis. Liver stiffness as determined by transient elastography significantly decreased from baseline to FUP at 24 weeks (median values 18 and 9.2 KPa, respectively; *P* = .011).

Median duration of treatment in the study population—as programmed by clinicians before starting DAA and reported in medical charts—was 16 weeks, which was not statistically different from the duration that could be administered. More in detail, and not taking the aforementioned 2 patients who had to prematurely discontinue all treatments into account, the following therapy schedules were administered: 12 weeks (2 patients, 1%), 16 weeks (74 patients, 51%), 20 weeks (47 patients, 33%), and 24 weeks (22 patients, 15%). Tables 4 and 5 show the main characteristics of the patients when starting treatment, divided according to the aforementioned therapy durations. Marked differences were found between the different liver clinics in terms of treatment schedules. Basically, 3 centers adopted predetermined therapy durations (16 weeks for all subjects in the first 2 centers and 24 weeks in the third center), and the remaining 4 hospitals (which represent the vast majority of treatments: 76%) followed by some response-guided therapies. Among the latter group, 2 centers followed the rule of pre-setting all treatments to 24 weeks, then shortening them to 16 weeks in the case of rapid virological response (RVR) at TW4; the 2 other ones had a great variability of pre-set duration of therapy (16, 20, and 24 weeks) and then shortened or elongated the schedule, according to RVR and/or RBV full dosage maintaining.

### Virological Outcomes

In Tables 2 and 3, the principal virological parameters of patients during and after antiviral treatment are reported. Intention-to-treat (ITT) SVR12 was 95.9%, which raised to 97.1% in a PP analysis. The only 4 patients who did not reach SVR after completing a full course of therapy experienced a virological relapse.

Various analyses were performed to test if there were any significant differences between the various treatment durations, in particular, to explore if there was a propension of the prescribing physicians to treat the more severe subjects with longer durations. Tables 4 and 5 explore the differences between the various durations (single or grouped); Table 6 focuses on the patients receiving 12 weeks of treatment in comparison with those receiving longer treatments. As MELD scores appeared as the only relevant determinant of liver function to possibly differ between the various treatment durations, further comparison was conducted between the 12-week and the 20- or 24-week schedules, but in both cases, no significant differences could be found (*P* = .93 and .96, respectively).

Duration of therapy (categorized as <20, 20, and 24 weeks) was not statistically associated with SVR, both in ITT and PP (Table 6 Panel C) analyses. Moreover, no significant differences were found with respect to the different virological outcomes in median age, transient elastography value, platelet count, basal HCV-RNA level, and the frequencies of previous PEG-IFN treatment status or need to reduce RBV dosage during treatment. Instead, median basal Child–Pugh and MELD scores were statistically different between patients who achieved SVR or not (*P* = .03 for both).

Multivariate analysis failed to identify possible independent predictors of virological outcomes. The set of covariates was selected a priori based on a consensus of clinical expertise and included the most well-established baseline covariates associated with SVR: sex, age, body mass index, albumin, platelet count, total bilirubin, hemoglobin, HCV-RNA, a history of antiviral treatment, treatment duration, and Child–Pugh and MELD scores.

## Discussion

This real-life study—which, although not randomized and with many potential biases, has the merit of having been focused exclusively on a particular, very homogeneous subset of patients (i.e., all HCV GT2-infected subjects with very advanced liver disease)—confirmed that SOF plus RBV led to SVR rates higher than 95% both in PP and ITT analyses, with good tolerability, both in naïve and experienced subjects. These data are slightly higher than most clinical trials and real-life records analyzing cirrhosis^4,7,20-21^ and are similar only to those reported from the large multi-center prospective PITER study.^22^ It is noteworthy that the second choice in the order of preference was indeed 20 weeks (32%), although this duration was not tested in the main clinical studies available in the literature, except the previously mentioned Italian experience.^17^ The 12-week schedule, presumably representing the most widely validated regimen, was adopted in our local experience by only a very small minority of clinicians, probably because they feared a possible suboptimal efficacy in patients with advanced fibrosis. In our study, we could not show any differences in SVR rates between different therapy durations, similarly to what has been described by Mangia et al^17^ who, however, focused only on 16- and 20-week durations. This is due to the fact that our study was not powered for these subgroup analyses and the different treatment duration groups were not matched. As recently demonstrated, due to the extremely high rates of SVR attainable with this regimen, the comparison, for instance, of 16 with 20 weeks of treatment would have required the enrolment of more than 700 subjects per arm.^17,23^ As a consequence of what has just been reported, it is evident from our work that in the ultimate end, the different clinician behaviors with regard to treatment were not crucial for the virological outcomes. Although at multivariable logistic regression analysis, no independent predictors of response could be found, and higher degrees of hepatic failure as defined by Child–Pugh or MELD scores were observed in patients with unfavorable virological responses, as suggested also by others.^24^

Another important issue of this study was to clarify if there was a potential bias in prolonging treatment in patients with more severe diseases. With all the aforementioned power limitations, no determinants of liver function showed significant differences between the subjects receiving 12 weeks of treatment in comparison with those receiving longer schedules. So it was not possible to demonstrate a prescription bias. This obviously may also be due to the fact that ours was a very homogeneous casuistry.

The safety profile was mainly consistent with RBV-related known side effects and, as previously reported, the most common AEs, including anemia, were similar to other reports.^25-27^ Only a single, severe AE was attributed by the prescribing physician to SOF. This was a case of acute kidney injury in a patient with no particular risk factors for nephropathy. While deterioration of renal function is a well-known SOF side effect in patients with already-impaired renal function,^28^ similar reports for DAA mono-therapy with SOF are only anecdotal.^29^ It is noteworthy that ours was a casuistry of elderly patients (mean age: 71 years, 42%** —**75 years) with, as expected, a high burden of comorbidities and co‐medications but with a low incidence of AEs (although higher than with RBV-free treatments), similar to that reported in the major clinical studies on the aged population.^30-33^

With regard to the reported high mean age of the studied population—which in any case represents the paradigm of most case series of cirrhotic patients currently treated at least in the Western world—some further considerations can be made. While DAA treatment—including SOF plus RBV—may seem, at a first glance, to be feasible in virtually all patients regardless of age and comorbidities, the question arises whether old patients should always be considered for antiviral therapy. On the one hand, progression to cirrhosis has been shown to be an age-dependent process.^34^ However, given the high costs of current DAA regimens, treatment priority should clearly be given to patients with advanced liver disease, whereas treatment is not recommended in patients with limited life expectancy.^35^ Obviously, the decision to treat elderly patients or not is then greatly influenced by local guidelines and/or reimbursement policies, as well as societal considerations. In this study, all of the studied patients had cirrhosis by inclusion criteria, so the main driver for treatment initiation was not the prevention of fibrosis progression but, most likely, of further liver damage with its associated detrimental consequences. Other possible factors that generally can justify the decision to treat older patients can be concomitant patient psychological distress and impaired quality of life due to debilitating fatigue, in addition to the presence of possible extrahepatic manifestations which have been demonstrated to increase with age.^36,37^ However, all these aspects were not formally investigated in the present research, and this may represent a possible study limitation.

In the patient series, Child–Pugh and MELD scores did not show a significant improvement after antiviral treatment, similarly to what has been observed by others.^38^ This again may be due to the sample size and also to the relatively short-term FUP and to the fact that only 9 patients had baseline hepatic decompensation. In any case, hepatic transient elastography ameliorated at FUP, as previously reported by other groups.^39^

Thus, although SOF use as DAA monotherapy for HCV GT2 in combination with RBV is no longer recommended by most Western guidelines, our study confirms, albeit a posteriori, the validity of the registration trials and the scientific rationale of its current use. We can even speculate that SOF plus RBV may still be a valid option against the “easy” villain HCV GT2.^40^ In any case, as confirmed in our real-world study, it is essential that the treatment duration always exceeds 12 weeks, as there is now solid and incontrovertible evidence that 12 week-courses of SOF plus RBV are associated to lower SVR rates than expected according to clinical trials.^10^ In this regard, although the onset of anemia was—at least in our research—not associated with the overall duration of treatment, it is reasonable to postulate that shorter treatment durations against GT2—as it is now possible with the newer RBV‐free regimens—can represent an advantage not only in SVR achievement but also in terms of tolerability and adherence (and, potentially, overall cost reduction) and should therefore be preferred, particularly, in the elderly which was represented, as previously described, in most of our case series. So—bearing these obvious limitations in mind—the present and future location of this treatment is to be sought, especially when access to the aforementioned more potent pan-genotypic regimes is not possible. Paradigmatic from this point of view is what is reported in Asian-Pacific guidelines, which state that the penetration of the newer IFN-free therapies into standard management plans of many countries has been very slow despite outstanding responses to therapy, mainly due to economic restraints.^3^ In our opinion, therefore, there is still room for such a therapeutic regimen, that is in any case cheaper than the other more recent DAA combinations. This should help to speed up the definitive closure of the IFN era, preventing the otherwise predicted path toward the HCV-related peak of population morbidity and mortality.

## Figures and Tables

**Table 1.** Main Baseline Demographic and Clinical Features of the Studied Population

Patient Characteristic	Value	
A. Total Population (n = 147)	B. Treatment Completers (n = 145)
Male sex, n	71 (48)	69 (48)
Age, years	74 (44-87)	73 (44-87)
Body mass index, kg/m^2^	25.4 (17.1-40)	25.2 (17.1-38.9)
HCV RNA, ×10^3^ IU/mL	865 (9-85200)	850 (13-85200)
Child–Pugh score	5 (5-8)	5 (5-7)
Child–Pugh class: A, B	138 (94), 9 (6)	144 (99), 1 (1)
MELD score	7 (5-13)	7 (6-13)
Basal transient elastography*, kPa	18.0 (10.1-75.0)	18.0 (10.1-75.0)
ALT, IU/L	69 (12-310)	69 (12-310)
Total bilirubin, mg/dL	0.9 (0.3-2.0)	0.8 (0.3-1.9)
International normalized ratio, units	1.1 (0.9-1.6)	1.1 (0.9-1.6)
Platelets, ×10^9^/L	135 (38-327)	135 (38-327)
Creatinine, mg/dL	0.69 (0.43-1.3)	0.8 (0.5-1.3)
eGFR^†^, mL/min	75 (46-146)	75 (46-146)
Stage of renal function, n for 1, 2, 3	34 (23), 72 (49), 41 (28)	34 (24), 70 (48), 41 (28)
Albumin, g/dL	3.8 (2.9-4.6)	3.8 (2.9-4.6)
Hemoglobin, g/dL	13.5 (9.2-17.1)	13.6 (9.2-17.1)
- Male	14 (9.2-17.1)	14 (9.2-17.1)
- Female	13.4 (10.2-15.3)	13.4 (10.2-15.3)
Baseline anemia, n for total, M, F	35 (24), 16 (23), 19 (25)	33 (23)
Status of previous PEG-IFN treatment, n		
- Naïve	91 (62)	89 (61)
- Experienced: total, relapse, partial response, null response	56 (38), 18 (32), 5 (9), 33 (59)	56 (39), 18 (32), 5 (9), 33 (59)

Panel A: total sample and panel B: patients who could complete the planned treatment.

Data are presented as median (range) for continuous variables and as frequency (%) for categorical variables.

*Available for 130 patients; ^†^Estimated with CKD-EPI creatinine equation for persons between 18- and 70-year-old and with BIS 1 equation for subjects over 70 years of age.

HCV, hepatitis C virus; MELD, Model For End-Stage Liver Disease; ALT, alanine aminotransferase; eGFR, estimated glomerular filtration rate; PEG-IFN, pegylated-interferon; CKD-EPI, Chronic Kidney Disease Epidemiology Collaboration; BIS, Berlin Initiative Study.

**Table 2.** Main Clinical and Virological Parameters of Patients During Antiviral Treatment, n = 147

Parameter	Value
Ribavirin	
Initial dosage, mg/die	1000 (600-1400)
Initial dosage, mg/kg/die	13.7 (10.1-23.3)
- Patient weight < 75 kg (n = 91)	14.3 (10.3-23.3)
- Patient weight ≥ 75 kg (n = 56)	12.9 (10.1-16.0)
Patients who failed to take at least 80% of the total drug amount, n	67 (46)
- Dose reduction	60 (41)
- Discontinuation*	7 (5)
TW4	
- HCV RNA, ×10^3^ IU/mL	0 (0-0.06)
TW12	
- HCV RNA, ×10^3^ IU/mL	0 (0-0)
ET	
- HCV RNA, ×10^3^ IU/mL	0 (0-0.01)
- Child–Pugh score	5 (5-11)
- MELD score	7 (5-21)

Data are presented as median (range) for continuous variables and as frequency (%) for categorical variables.

*Composed of 2 patients who temporarily discontinued RBV, 3 patients who prematurely suspended all RBV, and 2 patients who completely stopped all medicines due to premature discontinuation of treatment (including 1 subject who died).

HCV, hepatitis C virus; TW4, therapy week 4; TW12, therapy week 12; ET, end of treatment; MELD, Model For End-Stage Liver Disease.

**Table 3.** Main Clinical and Virological Parameters of Patients After Antiviral Treatment, n = 147

**Parameter**	**Value**
FUP12	
- HCV RNA, ×10^3^ IU/mL	0 (0-28)
- Child–Pugh score	5 (5-8)
- MELD score	7 (5-11)
FUP24	
- HCV RNA, ×10^3^ IU/mL	0 (0-95)
- Child–Pugh score	5 (5-7)
- MELD score	7 (6-14)
- Transient elastography, kPa	9.2 (7.1-65.0)
- Patient status:	
- Alive, n	75 (99)
- Dead, n	1 (1)
Virological outcomes	
- SVR, n	141 (96)
- Relapse, n	4 (2)
- Null response, n	2 (1)
Treatment duration	
- As initially programmed by clinicians, weeks	16 (12-24)
- Effective duration, weeks	16 (5-24)

Data are presented as median (range) for continuous variables and as frequency (%) for categorical variables.

HCV, hepatitis C virus; FUP12, post-treatment follow-up at 12 weeks; FUP24, post-treatment follow-up at 24 weeks; MELD, Model For End-Stage Liver Disease; SVR, sustained virological response.

**Table 4.** Comparison of the Main Demographic and Clinical Features of the Patients Who Completed the Planned Treatment (n = 145), Divided According to the Single Different Treatment Durations

**Characteristics**	**Treatment Durations**				*P*
**12 weeks** **(**n **=** 2**)**	**16 weeks** **(**n **=** 74**)**	**20 weeks** **(**n **=** 47**)**	**24 weeks** **(**n **=** 22**)**
Male sex, n	2 (100)	32 (43)	21 (45)	14 (64)	**.02**
Age, years	52 (50-54)	74 (49-87)	73 (50-81)	73 (44-85)	**.03**
Body mass index, kg/m^2^	31.0 (28.9-33.1)	25.3 (17.3-35.7)	25.4 (17.1-38.9)	23.8 (20.7-28.1)	.81
HCV RNA, ×10^3^ IU/mL	2975 (1345-4183)	430 (9-7135)	979 (13-85200)	1313 (71-6790)	**.049**
Child–Pugh score	5 (5-5)	5 (5-7)	5 (5-7)	5 (5-7)	.07
MELD score	7 (7-8)	7 (6-13)	7 (6-13)	7 (6-12)	**.004**
Basal transient elastography, kPa	13.8 (12.9-14.8)	25.3 (17.3-35.7)	20.5 (10.1-75.0)	21.0 (11.9-63.0)	.17
Platelets, ×10^9^/L	129 (110-149)	139 (43-327)	134 (38-242)	148 (67-216)	.74
Albumin, g/dL	4.0 (3.9-4.1)	3.8 (2.9-4.5)	3.8 (3.3-4.6)	3.7 (2.9-4.2)	.88
Creatinine, mg/dL	0.7 (0.7-0.8)	0.7 (0.5-1.1)	0.8 (0.6-1.3)	0.8 (0.5-1.1)	.99
eGFR*, mL/min	72 (68-75)	77 (52-107)	75 (46-139)	74 (48-146)	.84
Hemoglobin, g/dL	13.8 (13.4-14.1)	14.0 (9.2-17.1)	13.4 (10.2-16.0)	12.7 (11.7-16.4)	.91
Baseline anemia, n	0 (0)	9 (12)	14 (30)	10 (45)	**.003**
Naive to previous PEG-IFN, n	1 (50)	41 (55)	29 (62)	18 (82)	.16
SVR, n	2 (100)	73 (99)	46 (98)	20 (91)	.18

Data are presented as median (range) for continuous variables and as frequency (%) for categorical variables. Bold text indicates a statistically significant difference with a *P*-value less than .05. Bold values denote statistical signiﬁcance at the *P* < .05 level.

*Estimated with CKD-EPI creatinine equation for persons between 18- and 70-year-old and with BIS 1 equation for subjects over 70 years of age.

HCV, hepatitis C virus; MELD, Model For End-Stage Liver Disease; Egfr, estimated glomerular filtration rate; PEG-IFN, pegylated-interferon; CKD-EPI, Chronic Kidney Disease Epidemiology Collaboration; BIS, Berlin Initiative Study; SVR, sustained virological response.

**Table 5.** Main Baseline Demographic and Clinical Parameters of the Patients Who Completed the Planned Treatment Grouped for 12 and 16 Weeks (Panel A) and 16, 20, and 24 Weeks (Panel B) Treatment Durations

**Characteristics**	**Treatment Durations**	
	**A. 12-16 Weeks**	**B. 16-20-24 Weeks**
**(**n **=** 76**)**	**(**n **=** 143**)**
Male sex, n	34 (45)	67 (47)
Age, years	74 (49-87)	74 (44-87)
Body mass index, kg/m^2^	25.4 (17.3-35.7)	25.2 (17.1-38.9)
HCV RNA, ×10^3^ IU/mL	502 (9-7135)	850 (9-85200)
Child–Pugh score	5 (5-7)	5 (5-7)
MELD score	7 (6-13)	7 (6-13)
Basal transient elastography, kPa	17.3 (11.5-34.8)	19.0 (10.1-75.0)
Platelets, ×10^9^/L	139 (43-327)	135 (38-327)
Albumin, g/dL	3.8 (2.9-4.5)	3.8 (2.9-4.6)
Creatinine, mg/dL	0.7 (0.5-1.1)	0.8 (0.5-1.3)
eGFR*, mL/min	75 (59-107)	75 (46-146)
Hemoglobin, g/dL	14.0 (9.2-17.1)	13.6 (9.2-17.1)
Baseline anemia, n	9 (12)	33 (23)
Naive to previous PEG-IFN treatment, n	42 (55)	88 (62)
SVR, n	75 (99)	139 (97)

Data are presented as median (range) for continuous variables and as frequency (%) for categorical variables.

*Estimated with CKD-EPI creatinine equation for persons between 18- and 70-year-old and with BIS 1 equation for subjects over 70 years of age.

HCV, hepatitis C virus; MELD, Model For End-Stage Liver Disease; eGFR, estimated glomerular filtration rate; PEG-IFN, pegylated-interferon; CKD-EPI, Chronic Kidney Disease Epidemiology Collaboration; BIS, Berlin Initiative Study; SVR, sustained virological response.

**Table 6.** Comparison of the Main Demographic and Clinical Parameters of Patients Who Completed the Planned Treatment. The Following Therapy Durations Are Considered: 12 Weeks Versus 16 Weeks (Panel A), 12 Weeks Versus Longer Schedules (Panel B), 12 and 16 Weeks Versus 20 Weeks Versus 24 Weeks (Panel C)

**Characteristics**	**Treatment Durations**		
**A. 12 Weeks Versus 16 Weeks,** *P*	**B. 12 Weeks Versus** **16, 20, 24 Weeks,** *P*	**C. 12-16 Weeks** **Versus 20 Weeks Versus 24 Weeks,** *P*
Male sex, n	.20	.22	.28
Age, years	**.02**	**.03**	.18
Body mass index, kg/m^2^	**.049**	.07	.94
HCV RNA, ×10^3^ IU/mL	**.03**	**.04**	<.005
Child–Pugh score	.14	.21	.071
MELD score	.54	.81	**.003**
Basal transient elastography, kPa	.06	.07	.70
Platelets, ×10^9^/L	.68	.84	.56
Albumin, g/dL	.11	.18	.83
Creatinine, mg/dL	.83	.75	.97
eGFR*, mL/min	.67	.58	.76
Hemoglobin, g/dL	.62	.89	.77
Baseline anemia, n	.78	.59	**.001**
Naive to previous PEG-IFN treatment, n	.70	.62	.08
SVR, n	.97	.95	.14

Bold values denote statistical signiﬁcance at the ***P*** < .05 level.

*Estimated with CKD-EPI creatinine equation for persons between 18- and 70-years-old and with BIS 1 equation for subjects over 70 years of age.

HCV, hepatitis C virus; MELD, Model For End-Stage Liver Disease; eGFR, estimated glomerular filtration rate; PEG-IFN, pegylated-interferon; CKD-EPI, Chronic Kidney Disease Epidemiology Collaboration; BIS, Berlin Initiative Study; SVR, sustained virological response.
